# Leg-through motion patterns during diaper wearing in older adults: a multicenter cross-sectional study

**DOI:** 10.1186/s12877-026-07149-8

**Published:** 2026-02-23

**Authors:** Soichiro Koyama, Kenji Iwata, Yusuke Nakamura, Ikuko Sako, Shigeo Tanabe, Yohei Otaka

**Affiliations:** 1 Faculty of Rehabilitation, School of Health Sciences, Fujita Health University, Toyoake, Aichi Japan; 2 Japan Pro-care sales head office, Chubu Branch, Unicharm Corporation, Nagoya, Aichi Japan; 3 Life Time Value Co-Creation Department, Global Research & Development Division, Unicharm Corporation, Toyohamacho, Kagawa Japan; 4 Department of Rehabilitation Medicine, School of Medicine, Fujita Health University, Toyoake, Aichi Japan

**Keywords:** Patient care management, Clothing, Motor skills, Long-term care, Activities of daily living

## Abstract

**Background:**

The use of diapers among older adults is increasing in aging societies. However, the optimal method of wearing them remains unknown. We aimed to explore procedural variations in leg-through motion during diaper wearing in older adults.

**Methods:**

A multicenter cross-sectional study was conducted in 19 facilities in Japan. Individuals aged 65–99 years in nursing homes/adult daycare facilities and able to maintain a sitting posture in a chair were recruited via convenience sampling between March 2024 and January 2025. Leg-through motion during diaper wearing was recorded with the participants seated in a chair. The motion patterns were visually classified according to predetermined criteria. The classification of the observed motion patterns was subjected to a simple descriptive analysis. The inter-rater agreement of the classifications between the two assessors was determined using Cohen’s kappa coefficient.

**Results:**

Among 149 older adults (mean [standard deviation] age: 87.0 [7.2] years), the motions “keep the foot off the floor and use both hands” and “keep feet on the floor and use both hands” collectively accounted for 65.7% of the initial leg motions. For the subsequent leg, three motions—“keep the foot off the floor and use contralateral hand,” “keep the foot off the floor and use ipsilateral hand,” and “keep the foot off the floor and use both hands”—accounted for a total of 59.1%. Furthermore, the leg-through motion patterns varied according to the participants’ usual means of mobility. The inter-rater agreement was good for the initial leg (kappa = 0.72) and subsequent leg (0.80).

**Conclusions:**

We classified the motion patterns of older adults during diaper wearing. Our results provide fundamental insights into appropriate care and rehabilitation interventions. Furthermore, these findings inform further hypothesis-driven research on diaper-wearing kinematics and support the development of effective strategies to enhance independence diaper use in older adults.

## Background

The number of older adults is increasing worldwide. By the mid-2030s, the number of adults aged ≥ 80 years is projected to reach 265 million, with a substantial increase in the number of those aged ≥ 65 years over the next 30 years [1]. An international cross-sectional study showed a high prevalence of incontinence among long-term care residents in East Asia [2]. In Japan, the prevalence rates of urinary, fecal, and double incontinence are 66.9%, 42.8%, and 41.1%, respectively [3]. Providing toileting assistance to older adults with incontinence is a particularly challenging task associated with increased caregiving time [4], decreased caregiver sleep time [5], increased perceived caregiver burden [6, 7], and decreased quality of life among older adults with incontinence [6]. Family caregivers often experience a greater burden than nursing home staff, and urinary/fecal leakage from incontinence products is a major burden [8].

Adult diapers are critical management tools for older adults with incontinence, particularly those experiencing functional limitations that impede independent toileting. Diaper use helps older adults maintain their privacy and dignity by managing incontinence, leading to improved daily social and familial activities and ultimately improving their quality of life [9]. Furthermore, diaper use has been linked to improved health-related quality of life and a reduced risk of pressure ulcers [10]. Specifically, adult diapers facilitate greater participation in outdoor activities for older adults with incontinence [11].

When older adults manage adult diapers independently, it further preserves their autonomy and dignity while reducing their caregiver dependency; however, research on the proper procedures for wearing these diapers is lacking. In particular, underwear-type adult diapers should be designed for independent wearing; however, many older adults and patients with physical disabilities struggle with independent diaper wearing, particularly the “leg-through” motion [12]. This difficulty may be due to age-related motor and cognitive decline, including reduced finger coordination [13, 14], limited flexion range of motion of the spine and hip joints [15, 16], and decreased equilibrium and balance ability [17]. All these factors may impair the ability to maneuver the diaper while maintaining a stable posture.

This study aimed to explore leg-through motion patterns among older adults in nursing homes or adult day care facilities. Understanding these motion patterns is crucial for enhancing independence in toileting, informing assistive product design, and bridging caregiving ergonomics with rehabilitation science. The present study is a foundational step toward developing effective training procedures for wearing adult diapers, ultimately aiming to enhance the independence and quality of life of older adults.

## Methods

### Study design and settings

This multicenter cross-sectional observational study was conducted in Japan between March 2024 and January 2025 at 19 facilities in 6 prefectures: 5, 5, 3, 3, 2, and 1 in Okayama, Kagawa, Aichi, Ehime, Hiroshima, and Chiba, respectively. The facilities were selected using convenience sampling. The types of facilities were as follows: eight fee-based homes for older adults with nursing care, four intensive care homes for older adults, two assisted living facilities, two long-term care health facilities, one group home, and two adult daycare facilities. This study was conducted in accordance with the principles of the Declaration of Helsinki and approved by the Ethics Review Committee of our university (No. HM 23–214). All participants provided written informed consent before participating in the study. In addition, this study report followed the STROBE (Strengthening the Reporting of Observational Studies in Epidemiology) reporting guidelines (https://www.strobe-statement.org/).

### Participants

Participants were recruited using convenience sampling. The researchers explained the outline of the study to the facility staff and used flyers displayed within the facility to recruit participants. The inclusion criteria were as follows: living in facilities for older adults or using long-term care services, aged 65–99 years, an adult diaper user in daily living, and able to maintain an independent sitting posture in a chair with a backrest and armrests. The exclusion criteria were as follows: individuals whose doctors had restricted hip or trunk flexion movements (e.g., those with artificial hips or lumbar compression fractures) and presence of cognitive impairment that was severe enough to prevent understanding of the study procedures or affect the ability to follow task instructions. This exploratory study aimed to characterize diaper-wearing motion classifications using descriptive statistics. Because leg-through motion patterns have not yet been established, a formal sample size calculation was not feasible. Therefore, participants were recruited from the accessible population during the study period to maximize sample size.

### Experimental procedure

The experiment was conducted within the facility used by the participants. The participants wore their usual clothing. Each participant sat on a chair with a backrest and arm support, with both feet on the ground. The seat height was adjusted to position the participants with hip and knee flexion at 90°. All participants performed the diaper-wearing task without caregiver assistance or guidance. They were instructed to wear the diaper at their own pace, as they normally would. Participants wore adult diapers (Lifree Usugata Keikai Pants, Unicharm Co. Ehime, Japan) in size M or L, selected according to body size. No additional instructions regarding specific motion techniques or time constraints were provided to ensure they performed their natural motion patterns. These standardized conditions were applied uniformly to all participants, regardless of care level. The participants were required to perform the diaper-wearing motion once. To record the entire leg-through motion of diaper wearing, three 11-inch iPad Pros (2nd generation, 1080p HD/30fps, Apple Inc., Cupertino, CA, USA) were used to capture two-dimensional video data (one frontal and two sagittal views).

### Expert-defined leg-through motions for diaper wearing

**Fig. 1:**
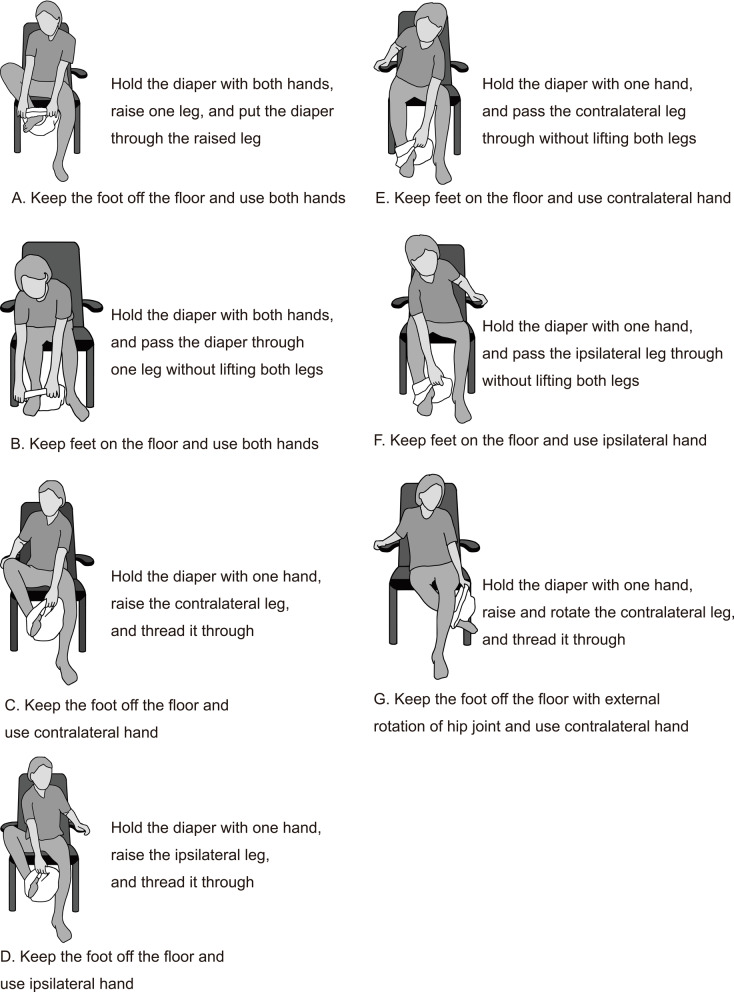
Definitions of leg-through motions for the initial leg

**Fig. 2:**
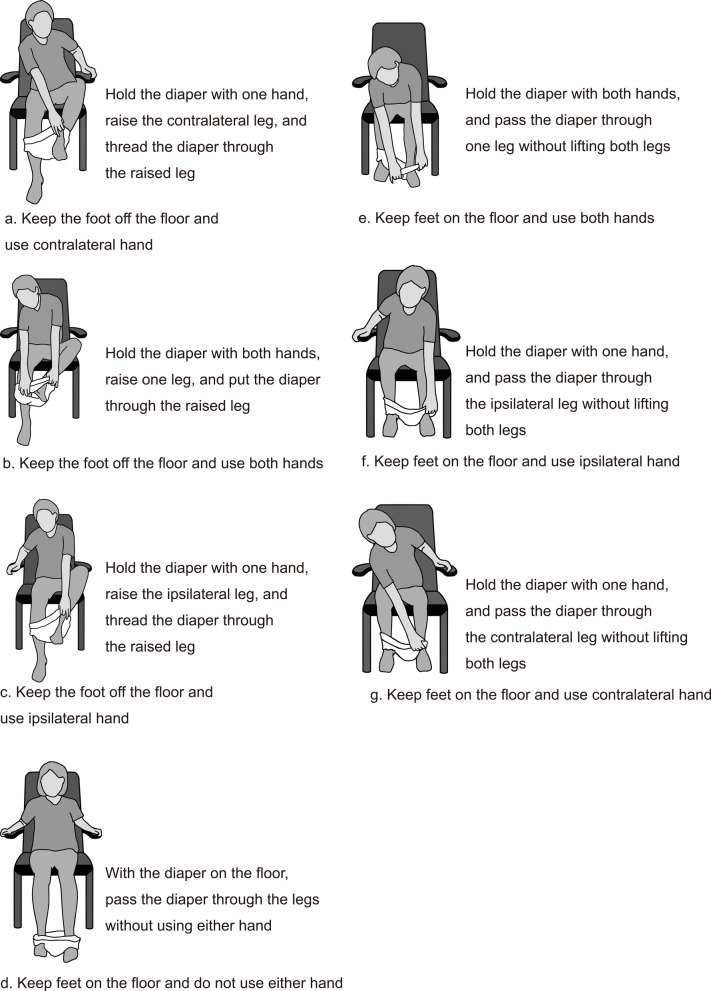
Definitions of leg-through motions for the subsequent leg

Prior to data acquisition, a panel of six experts, the authors of this paper, defined the leg-through motion patterns for diaper wearing by older adults. This expert group comprised one board-certified rehabilitation physician (YO, 26 years of clinical experience), three physical therapists (SK, 19 years; KI, 19 years; ST, 26 years of clinical experience), and two adult diaper developers (IS, 5 years; YN, 6 years of experience). Since the standard method for wearing a diaper involves sliding each leg into the diaper, one at a time, the process can be divided into two phases: the initial leg and subsequent leg phases. The experts defined seven distinct patterns for the initial foot insertion (Fig. 1) and subsequent foot insertion (Fig. 2), distinguished by foot positioning (on or off the floor), hand usage (both hands, contralateral, ipsilateral, or none), and specific joint motions, such as hip external rotation. These experts independently identified potential leg-through motion patterns based on their clinical observations and professional experience. Three face-to-face consensus meetings were subsequently held to discuss, refine, and finalize the classification categories. Final consensus was achieved when all six experts unanimously agreed on the motion pattern classification.

### Classification of participants’ motion patterns

Videos of leg-through motion patterns during diaper wearing were retrospectively classified by two assessors independently according to predetermined criteria. To ensure adequate assessor viewing, two video planes were used to document the quality of movements and positions. One of the assessors was a specialist in physical therapy (SK) with 19 years of clinical experience working with older adults. The other assessor was a staff member (IS) with 5 years of experience in the development of adult diapers. Both assessors were allowed to replay the video recordings at their discretion. Finally, discrepancies in the classification of leg-through motion patterns were addressed by discussions between the two assessors until an agreement was reached.

### Data collection

The following data regarding the characteristics of older adults were extracted from the care records: age (years), height, weight, sex, usual means of mobility, and care level in the Japanese long-term care insurance system. The usual means of mobility were classified as follows: independent walkers, assisted walkers (using walking aids or personal assistance), and wheelchair users. The care level was classified into seven categories, ranging from mild to severe disability, according to an individual’s activities of daily living and physical function [18]: Support Level 1 and Support Level 2 for individuals requiring light support, and Care Level 1 (lowest care need) through to Care Level 5 (highest care need, i.e. fully dependent) for individuals requiring care [19]. In addition, we assessed the grip strength in both hands to measure overall muscle strength [20].

### Statistical analysis

The participants’ characteristics are presented using usual descriptive statistics. Body mass index (BMI) was calculated as weight (kg) divided by height squared (m^2^). Continuous data are presented as mean (standard deviation [SD]). Categorical variables are expressed as frequencies and proportions. The classification of the observed motion patterns was subjected to a simple descriptive analysis. The leg-through motion patterns for the initial and subsequent foot are presented by the number of participants and their corresponding percentages. The association between leg-through motion patterns and the usual means of mobility was analyzed using Fisher’s exact test for each initial and subsequent leg. The inter-rater agreement for the visual classification of the participants’ motion patterns based on the predetermined criteria of the leg-through motion patterns was evaluated using Cohen’s kappa coefficients (k). Kappa values were interpreted as follows: very good agreement (≥ 0.8), good agreement (≥ 0.6 and < 0.8), and moderate agreement (≥ 0.4 and < 0.6) [21]. Statistical analysis was performed using Stata/SE 19.0 (StataCorp., College Station, TX, USA). Statistical significance was set at a p-value of 0.05.

## Results

### Participants

A total of 149 older adults met the inclusion criteria. The participants’ characteristics are presented in Table 1. Their mean age was 87.0 years (SD: 7.2), and 107 (71.8%) participants were female. BMI distribution showed that most participants (*n* = 103, 69.1%) fell within the normal range (18.5–24.9 kg/m²). The most frequent usual means of mobility was “assisted walkers” (69 participants, 46.3%), followed by “wheelchair users” (56 participants, 37.6%), and “independent walkers” (21 participants, 14.1%). Regarding care levels, the most frequent level was “Care level 2” (47 participants, 31.5%), followed by “Care Level 3” (38 participants, 25.5%) and “Care level 1” (38 participants, 25.5%). Over 80% of the participants were either required minimal to constant assistance in their daily activities. The average grip strength was 14.1 kg and 12.8 kg for the right and left hands, respectively, indicating a decline in muscle strength [22, 23].

**Table 1:** Participants’ demographics (*N* = 149)

Age, years	87.0 (7.2)
Height, cm	150.0 (9.9)
Weight, kg	48.4 (9.7)
Body mass index, kg/m^2^	
<18.5	28 (18.8)
18.5–24.9	103 (69.1)
25.0–29.9	17 (11.4)
≥30.0	1 (0.7)
Sex	
Female	107 (71.8)
Male	42 (28.8)
Usual means of mobility*	
Independent walkers	21 (14.1)
Assisted walkers (using walking aids or personal assistance)	69 (46.3)
Wheelchair user	56 (37.6)
Care level	
Support level 1	2 (1.3)
Support level 2	5 (3.4)
Care level 1	38 (25.5)
Care level 2	47 (31.5)
Care level 3	38 (25.5)
Care level 4	12 (8.1)
Care level 5	3 (2.0)
Not certified	4 (2.7)
Grip strength, KgF	
Right	14.1 (6.0)
Left	12.8 (6.0)

Values are presented as mean (standard deviation) or number of participants (%). *Values are missing for three participants

### Leg-through motion patterns during diaper wearing

Table 2 presents the percentages of leg-through motion patterns for both legs, based on the final consensus classification of two raters (SK and IS). For the initial leg, the most frequently observed motion was “keep the foot off the floor and use both hands” (43.6%), followed by the pattern of “keep feet on the floor and use both hands” (22.1%). For the subsequent leg, the most frequently observed movement was “keep the foot off the floor and use contralateral hand” (27.5%). The movement “keep the foot off the floor and use both hands” (19.5%) was the second most frequently observed type. The “Others” category included threading both legs simultaneously in each leg phase. For the initial leg, 12 participants were unable to perform the leg-through motions; for the subsequent leg, an additional 11 participants were unable to perform them. In other words, the 12 participants who were unable to perform the initial leg-through motions were included in the group of 23 who could not perform the subsequent leg-through motions.

**Table 2:** Participants’ leg-through motion patterns based on the final consensus classification by two raters

Patterns*	Number of participants (%)
Initial foot motion type	
A. Keep the foot off the floor and use both hands	65 (43.6)
B. Keep feet on the floor and use both hands	33 (22.1)
C. Keep the foot off the floor and use contralateral hand	22 (14.8)
D. Keep the foot off the floor and use ipsilateral hand	3 (2.0)
E. Keep feet on the floor and use contralateral hand	2 (1.3)
F. Keep feet on the floor and use ipsilateral hand	1 (0.7)
G. Keep the foot off the floor with external rotation of hip joint and use contralateral hand	0 (0)
H. Others	11 (7.4)
I. Not independent	12 (8.1)
Subsequent foot motion type	
a. Keep the foot off the floor and use contralateral hand	41 (27.5)
b. Keep the foot off the floor and use both hands	29 (19.5)
c. Keep the foot off the floor and use ipsilateral hand	18 (12.1)
d. Keep feet on the floor and do not use either hand	11 (7.4)
e. Keep feet on the floor and use both hands	10 (6.7)
f. Keep feet on the floor and use ipsilateral hand	7 (4.7)
g. Keep feet on the floor and use contralateral hand	2 (1.3)
h. Others	8 (5.4)
i. Not independent	23 (15.4)

*Patterns are ranked in descending order of frequency

### Relationship between leg-through motion patterns and the usual means of mobility

**Fig. 3:**
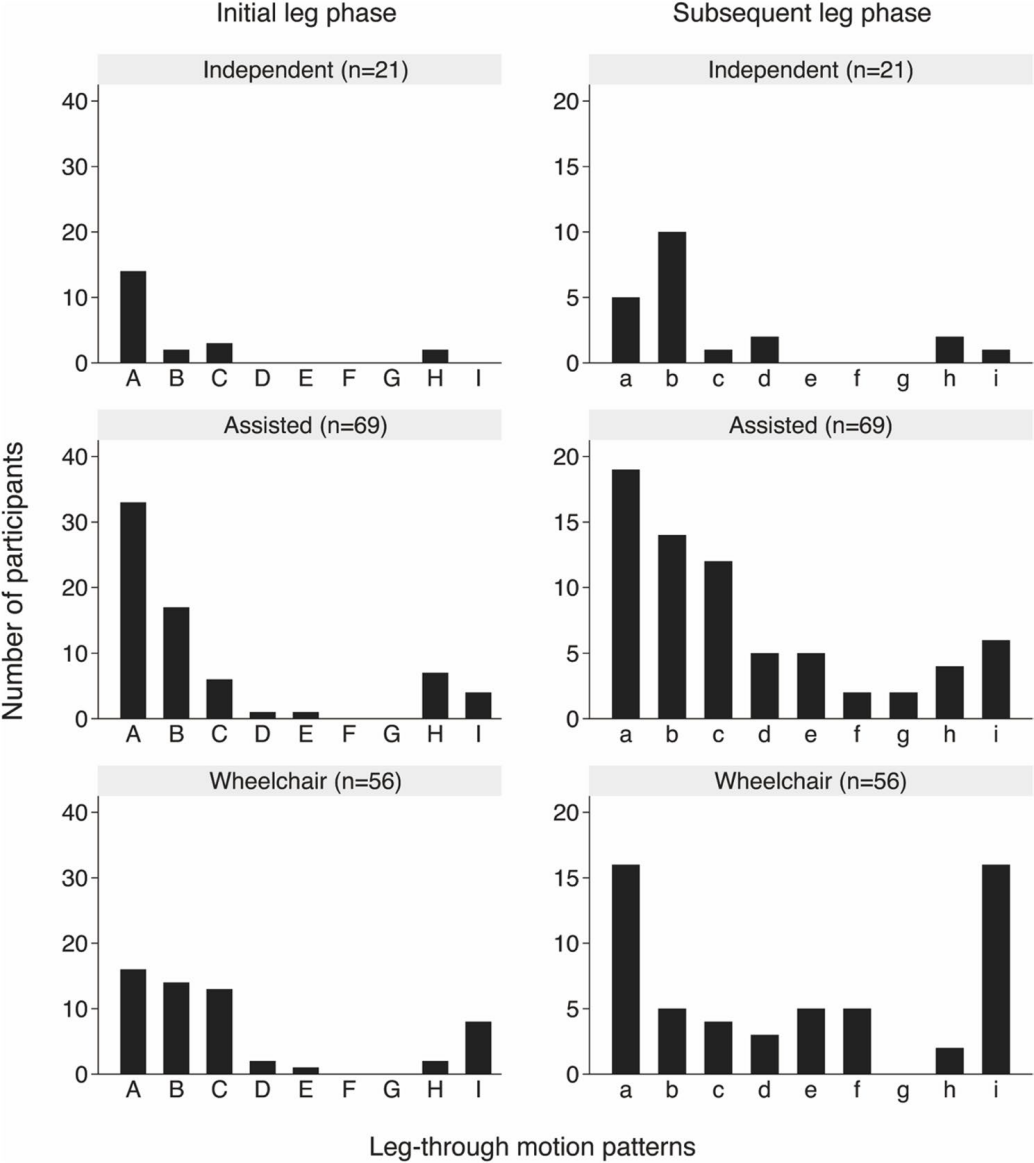
Leg-through motion patterns stratified by the usual means of mobility

Leg-through motion patterns varied significantly based on the participants’ usual means of mobility, with statistically significant associations confirmed by Fisher’s exact tests for both the initial leg (*p* = 0.039) and subsequent leg (*p* = 0.007). For the initial leg, independent walkers primarily used pattern A (keep the foot off the floor and use both hands), accounting for 66% (14/21) of this group. Wheelchair users showed the following distribution of pattern: pattern A was observed in 29% (16/56), pattern B (keep feet on the floor and use both hands) in 25% (14/56), and pattern C (keep the foot off the floor and use contralateral hand) in 23% (13/56). Notably, 14% (8/56) of wheelchair users were unable to perform independent leg-through motion during diaper wearing. For the subsequent leg, independent walkers most frequently used pattern b (keep the foot off the floor and use both hands), accounting for 48% (10/21), similar to the dominant pattern observed for the initial leg. Wheelchair users most often employed pattern a (keep the foot off the floor and use contralateral hand) at 29% (16/56); however, other patterns were also observed. The proportion of wheelchair users unable to complete the motion independently increased substantially to 29% (16/56) during the subsequent leg phase (Fig. 3). 

### Inter-rater agreement

Table 3 shows the visual classification of the participants’ leg-through motion patterns. The inter-rater agreement of visual classification showed good agreement for the initial leg (k [95% confidence interval] = 0.72 [0.64–0.80], *p* < 0.001) and subsequent leg (0.80 [0.72–0.87], *p* < 0.001).

**Table 3:** Leg-through motion patterns by two assessors

	Patterns*	Assessor 1 Number of participants (%)	Assessor 2 Number of participants (%)
Initial foot	A. Keep the foot off the floor and use both hands	68 (45.6)	67 (45.0)
	B. Keep feet on the floor and use both hands	38 (25.5)	33 (22.1)
	C. Keep the foot off the floor and use contralateral hand	23 (15.4)	22 (14.8)
	D. Keep the foot off the floor and use ipsilateral hand	4 (2.7)	3 (2.0)
	E. Keep feet on the floor and use contralateral hand	1 (0.7)	2 (1.3)
	F. Keep feet on the floor and use ipsilateral hand	0 (0.0)	1 (0.7)
	G. Keep the foot off the floor with external rotation of hip joint and use contralateral hand	0 (0.0)	0 (0.0)
	H. Others	5 (3.4)	11 (7.4)
	I. Not independent	10 (6.7)	10 (6.7)
Subsequent foot	a. Keep the foot off the floor and use contralateral hand	45 (30.2)	41 (27.5)
	b. Keep the foot off the floor and use both hands	34 (22.8)	25 (16.8)
	c. Keep the foot off the floor and use ipsilateral hand	17 (11.4)	22 (14.8)
	d. Keep feet on the floor and do not use either hand	12 (8.1)	11 (7.4)
	e. Keep feet on the floor and use both hands	11 (7.4)	10 (6.7)
	f. Keep feet on the floor and use ipsilateral hand	4 (2.7)	7 (4.7)
	g. Keep feet on the floor and use contralateral hand	2 (1.3)	2 (1.3)
	h. Others	1 (0.7)	8 (5.4)
	i. Not independent	23 (15.4)	23 (15.4)

*Patterns are ranked in descending order of frequency

## Discussion

We investigated the leg-through motion patterns involved in diaper wearing by older adults. The observed leg-through motion patterns almost fitted the predefined motion patterns. The leg-through motion patterns included six patterns for the initial leg and seven patterns for the subsequent leg. Furthermore, the classification of the leg-through motion patterns varied according to the participants’ usual means of mobility. To the best of our knowledge, this is the first study to explore the leg-through motion patterns of diaper wearing in an older adult population. Despite the fact that toileting difficulties are well-documented as a major source of caregiver burden [24], no study has focused on the process of wearing diapers. Our findings may offer valuable knowledge for appropriate care and rehabilitation interventions for individuals who need diapers.

The two bilateral hand manipulation strategies—“keep the foot off the floor and use both hands” and “keep feet on the floor and use both hands”—accounted for 65.7% of initial leg-through motion patterns (*n* = 98). This indicates that approximately two-thirds of the participants used both hands to manipulate the diaper during the initial foot insertion. In subsequent leg motion patterns, three predominant strategies—“keep the foot off the floor and use contralateral hand” (27.5%), “keep the foot off the floor and use both hands” (19.5%), and “keep the foot off the floor and use ipsilateral hand” (12.1%)—collectively accounted for 59.6% (*n* = 88).

During initial leg insertion, most participants used both hands to maneuver the diaper. Because it was necessary to sufficiently widen the leg openings and guide the initial leg smoothly into the diaper, this bilateral manual pattern may appear to be the standard approach. Indeed, independent walkers predominantly performed pattern A which involves manipulating the diaper with both hands to spread the leg hole opening for insertion by the initial leg. Present results suggested that pattern A may represent an efficient leg-through pattern for individuals with sufficient function. On the other hand, individuals with specific physical limitations that hinder this approach need to adopt a different leg-through motion pattern. In the present study, some participants used only one hand during the initial leg insertion. This one-handed maneuver can be considered a compensatory strategy to overcome the difficulty of reaching the diaper opening with their toe while holding the diaper open with both hands. This difficulty might be due to functional restrictions in spinal or hip range of motion or to sitting balance deficits. The participants with balance deficits may have been anxious about maintaining their seated position when inserting the initial leg, and they used the armrest to maintain postural stability while manipulating the diaper with one hand.

Approximately 60% of participants used a “keep-foot-off-floor” technique for subsequent leg insertion, which may reflect sufficient seated stability. In fact, independent walkers predominantly used pattern b, which involved manipulating the diaper with both hands to spread the leg hole opening for insertion by the subsequent leg while keeping foot-off without hand support. On the other hand, wheelchair users demonstrated pattern a, which involves manipulating the diaper with one hand while keeping foot-off with hand support. Thus, pattern a may reflect individualized adaptations to their functional limitations on seated stability.

Interestingly, the most prevalent “keep-foot-off-floor” approach was maintaining the subsequent leg off the floor while using the contralateral hand rather than the ipsilateral hand for diaper manipulation. For individuals with no functional decline, it seems reasonable to pull the diaper caught on the initial leg with the opposite hand (i.e., the ipsilateral hand of the subsequent leg) and then pass the leg through. Individuals should use their ipsilateral hand to widen the diaper opening, ensuring sufficient hip flexion and minimal hip external rotation to avoid interference between the leg and hand. If this movement is not possible, the opposite hand must be used. Furthermore, if lifting and holding the subsequent leg is challenging, it must be supported with the ipsilateral hand. Some participants even held the subsequent leg with the ipsilateral upper limb to maintain hip flexion, while managing the diaper with the opposite upper limb. This compensatory pattern may suggest restrictions in hip flexor strength and range of motion. Weakness in the hip flexors can make it challenging to sustain leg elevation, and a restricted range of motion often necessitates the observed hip external rotation and abduction during flexion. Participants who used both hands to manipulate the diaper likely maximized the opening width to ease the insertion of the subsequent leg, thereby compensating for limited leg movement.

This study used an expert-defined leg-through motion classification. The visual classification of the participants’ leg-through motion patterns demonstrated acceptable inter-rater agreement. Cohen’s kappa coefficients revealed good agreement for the initial leg (k = 0.73) and subsequent leg (k = 0.80). These findings support the reliability of the predetermined criteria for visual classification in this study. Although visual classifications demonstrated acceptable inter-rater agreement, the present motion pattern classification was developed by a relatively small panel of six experts, which may have affected the diversity of perspectives and comprehensiveness of the classification system. To reduce potential professional bias and enhance the generalizability of the present findings, future research should verify validity and reliability using different participants, different raters (e.g., nurses, occupational therapists, and caregivers), and various clinical settings.

In this study, most of the motion patterns fit into the predefined motion patterns. However, the comprehensiveness of motion patterns must be interpreted with caution. First, the motion patterns classified in this study cannot be completely ruled out that rare patterns, i.e., those not observed in all 149 participants (< 1%), remain unidentified. In addition, approximately 5–7% of the participants were classified as “Others”. In this category, we observed a bilateral simultaneous leg-through motion pattern. In this pattern, the participants first lifted both toes off the floor while keeping both heels on the floor. Then, they inserted both toes into the leg openings of the diaper with both hands, followed by both heels. For older individuals with sufficient trunk flexibility to reach the floor with their hands in a sitting position, this motion may serve as a safe compensatory strategy, as both feet remain in contact with the floor during diaper wearing. The presence of the “Others” pattern suggests that our classification was not fully exhaustive; therefore, future studies should consider including bilateral simultaneous patterns as distinct classifications. Second, one predefined pattern (hip external rotation with hand support) was not observed (0%). Although this pattern is theoretically effective for individuals with restricted trunk or hip flexion, its absence in our cohort is likely attributable to the exclusion criteria, which omitted individuals with such physical restrictions for safety reasons. Therefore, while this pattern may not be common among older population without functional restrictions in trunk or hip flexion, it might be observed in clinical populations characterized by functional restrictions.

Further research on the relationship between physical function and motion patterns in older adults can help advance research on independent diaper wearing in older adults. Aging is associated with declines in the range of reach from a sitting position [25], and various sensory systems, including the vestibular, visual, and somatosensory systems, as well as the musculoskeletal system, which are associated with changes in muscle strength and joint range of motion [26–29]. Future studies should incorporate comprehensive quantitative physical function assessments, such as the range of joint motion, trunk control test, and timed up and go test, to clarify their relationships between specific physical abilities and the observed motion patterns. Furthermore, detailed motion analyses are needed to obtain data on the kinematic and kinetic aspects of motion that would be useful in clinical practice. Understanding the kinematic and kinetic aspects of diaper wearing is useful for goal-oriented rehabilitation and for developing training methods for diaper users and/or individuals who are unable to change their own diapers. In addition, given that diaper-wearing involves stepwise reasoning and spatial awareness, variations in cognitive abilities may have influenced the selection of motion patterns and task performance. Future studies should incorporate validated cognitive screening to systematically evaluate the relationship between cognitive status and leg-through motion patterns.

### Strengths and limitations

The strength of this study lies in the recruitment of participants from 19 diverse older care facilities across Japan, minimizing facility-specific influences on the results. Inter-rater agreement was evaluated using recorded data to ensure consistent viewing angles and eliminate assessment differences caused by various observer positions. Motion patterns were systematically defined in collaboration with rehabilitation physicians, physical therapists, and adult diaper developers, enabling the appropriate classification of observed behaviors from a clinical viewpoint.

This study has certain limitations. The participating facilities were selected using a convenience sampling method based on existing relationships with the authors. Hence, the possibility of selection bias cannot be ignored, as facilities that agreed to participate may differ from others in terms of organizational characteristics, staff training, and quality of care. Therefore, the results of this study may not be generalizable to all other facilities. Additionally, participants wore diapers over their usual clothing rather than directly on their skin. This deviation from typical diaper use conditions may have affected leg-through motion patterns such as restricted the range of motion on the hip and trunk joints, altered tactile feedback, and visual information during the actual leg-through motion pattern. Additionally, the use of identical underwear-type diapers may have influenced the results, as diaper characteristics (material softness, abdominal circumference, elasticity, and leg hole size) could affect movement patterns. These findings should be interpreted with caution when applying them to other types of diapers.

## Conclusions

To our knowledge, this is the first study to examine leg-insertion motions while wearing a diaper in older adults. This study provides foundational observational evidence of diverse motion patterns during diaper wearing that may inform future hypothesis-driven research on rehabilitation strategies to enhance independence diaper use in older adults. Adult diaper leg-insertion requires downward-reaching movements from a sitting position to access the lower extremities. Since sensory and motor functions decline with aging, future research should clarify the relationship between physical function and leg-insertion patterns by focusing specifically on downward-reaching abilities from sitting positions.

## Data Availability

The data that support the findings of this study are not publicly available owing to the sensitive nature of the data; however, they are available from the corresponding author upon reasonable request.

## Abbreviations

BMI: Body mass index
SD: Standard deviation
